# Generation of CRISPR-Cas9-mediated knockin mutant models in mice and MEFs for studies of polymorphism in clock genes

**DOI:** 10.1038/s41598-023-35203-7

**Published:** 2023-05-19

**Authors:** Kwangjun Lee, Choogon Lee

**Affiliations:** Program in Neuroscience, Department of Biomedical Sciences, College of Medicine, Florida State University, 1115 West Call Street, Tallahassee, FL 32306 USA

**Keywords:** Circadian mechanisms, CRISPR-Cas9 genome editing

## Abstract

The creation of mutant mice has been invaluable for advancing biomedical science, but is too time- and resource-intensive for investigating the full range of mutations and polymorphisms. Cell culture models are therefore an invaluable complement to mouse models, especially for cell-autonomous pathways like the circadian clock. In this study, we quantitatively assessed the use of CRISPR to create cell models in mouse embryonic fibroblasts (MEFs) as compared to mouse models. We generated two point mutations in the clock genes *Per1* and *Per2* in mice and in MEFs using the same sgRNAs and repair templates for HDR and quantified the frequency of the mutations by digital PCR. The frequency was about an order of magnitude higher in mouse zygotes compared to that in MEFs. However, the mutation frequency in MEFs was still high enough for clonal isolation by simple screening of a few dozen individual cells. The *Per* mutant cells that we generated provide important new insights into the role of the PAS domain in regulating PER phosphorylation, a key aspect of the circadian clock mechanism. Quantification of the mutation frequency in bulk MEF populations provides a valuable basis for optimizing CRISPR protocols and time/resource planning for generating cell models for further studies.

## Introduction

The prokaryotic defense system CRISPR-Cas provides adaptive immunity against foreign DNA molecules and has been converted to a revolutionary genome editing tool^1–3^. The CRISPR-Cas9 system from *Streptococcus pyogenes* is the most widely used because of its efficiency and simplicity^4^. The system requires two components: the Cas9 nuclease and a single guide RNA (sgRNA) which targets the nuclease to a specific genomic locus based on base pairing at a 20-nt target sequence. The sgRNA-guided Cas9 generates a double strand break (DSB) in the target site, which may be repaired in one of two different ways. The first is random insertion/deletion (indel) mutagenesis which occurs when the DSB is repaired by the error-prone non-homologous end-joining (NHEJ) pathway, resulting in a knockout allele due to frameshifting or indels of amino acids (AAs) due to in-frame nucleotide indels^4^. The second is precise allele editing through the high-fidelity homology-directed repair (HDR) pathway based on a donor or repair template, which usually occurs less frequently than NHEJ^5,6^. More versatile than other genetic approaches such as siRNA and transgene expression, and more efficient than older homologous recombination methods^7,8^, CRISPR is becoming a more and more common method to modulate a gene to interrogate its function or for medical applications^9,10^.

CRISPR technology is rapidly evolving but still has many limitations. If target cells do not proliferate or are not easily transfected or electroporated with plasmids, clonal selection and expansion from the treated heterogenic population may not be possible or practical. CRISPR viral vectors have been employed to try to overcome these problems^11–16^. Clonal selection and expansion may not be required if the transduction efficiency and expression of the viral vectors are close to 100% and sgRNA efficiency is very high^17^. Several strategies have been developed to enhance HDR efficiency by activating HDR-related proteins or inhibiting NHEJ-related proteins^18–20^. HDR donor templates also affect the efficiency significantly. Various lengths, chemical modifications, donor strand preference and symmetry of homologous arms have been tested^4,21–23^. Although results vary significantly in these studies, it seems that asymmetric single stranded oligodeoxynucleotides (ssODNs) with phosphorothioate bonds from the non-targeting strand are most effective as HDR donor templates.

The circadian clock drives daily rhythms in behavior and physiology^24–28^, and dysfunction or disruption of the clock has been implicated in diverse disease states including sleep disorders^29–33^. Decades of prior work have revealed that the clock is built on a core feedback loop that is cell autonomous, involving transcriptional and post-translational regulation of the redundant pacemaker *Period* (*Per*) genes *Per1* and *Per2*^34,35^. Because the circadian clock is cell autonomous, genetic disruptions of the clock manifest similar phenotypes at the behavioral and cellular levels, and cell culture has proven to be a valuable and valid platform for characterizing the molecular biology of circadian rhythms^36–38^. The endogenous clocks of cultured cells—including mouse embryonic fibroblasts (MEFs) and human U2OS cells—can be precisely measured in real time by introducing a luciferase (Luc) reporter gene under control of a clock promoter^36,38–40^. Across numerous studies, such cells have served as functional models for in vivo circadian clocks, and results have been consistently validated in live animal models. Cell culture models are not only less resource-consuming, but also more easily manipulated by chemicals and transgenes, which makes the cell models more suitable for mechanistic studies.

Historically, manipulation of endogenous clock genes in cell culture models suffered from technical limitations. Many genetic cell models required first developing mutant mouse models from which cells were then harvested. For example, mice with the *mPer2-Luc* knockin gene were crossed with mice with other mutations, backcrossed as needed, and MEFs were obtained from the resulting transgenic/mutant offspring^41–43^. Recent developments in genome editing have created new opportunities for generating cell culture models without first generating mutant mice. Several studies including ours demonstrated that clock genes can be knocked out efficiently in culture using CRISPR^17,44–47^. To our knowledge, however, there are no HDR-mediated mutations made in clock genes in MEFs by CRISPR. Because many mutant and transgenic mouse models including *mPer2-Luc* knockin are already available, it would be advantageous to implement direct genome editing in MEFs derived from these existing genetic mouse models.

In this current study we generated two CRISPR-mediated SNP mutations in mice and MEFs and quantified and compared the efficiency between mice and MEFs. Digital PCR can be a powerful tool for genotyping of CRISPR mutant mice when indels are too large to be detected by conventional PCR-based genotyping. When HDR-mediated mutations are generated in cells, we show that digital PCR is a simple yet powerful tool to accurately quantify the frequency of the mutations in the heterogenous cell population. Measuring the frequency of the mutations would directly inform optimization of CRISPR procedures and amounts of effort necessary for downstream clonal isolation.

## Results

### SNP mutations in *mPer* genes are generated efficiently in mice by CRISPR-Cas9

Although key circadian parameters of the clock seem to be encoded in the PERIOD (PER) protein, it is little understood how 24 h time cues are generated by the regulation of PER. As with most other proteins, PER has a modular structure with multiple domains, including the PAS domain, CRY-binding domain (CBD), and CK1-binding domain (CKBD) (Fig. S1). Although PAS is known as a homo or hetero-dimerization domain and conserved from plants to animals as an essential timing device^48,49^, its role in mammalian clocks is little studied. To understand how the PAS domain contributes to rhythm generation of PER at posttranslational levels, we decided to disrupt the main function of PAS, homodimerization, in MEFs and mice.

Because the dimer structure of PER and PER PAS domains has been solved at high resolution by X-ray crystallography^50,51^, we targeted critical motifs and amino acid (AA) residues for dimerization based on these available data. According to the structural studies, motifs containing PER1-W448 and PER2-W419 AA are most critical for dimerization (Fig. S1). Tryptophan to glutamate mutations (PER1 W448E and PER2 W419E) have been suggested to be most disruptive to the hydrophobic interaction-mediated dimerization; we therefore planned to generate these mutations using CRISPR for functional studies.

Before we initiated the project in mouse models, feasibility of the project was tested in a clock cell model U2OS which has been proven to have a functional clock and to be amenable to CRISPR genome editing^17,47,52^. When the motifs harboring W448 in PER1 and W421 in PER2 (conserved residues in human PER proteins) were targeted by CRISPR, diverse indel mutations were generated including in-frame mutations leading to deletion of several AAs (Fig. S2). Interestingly, all of the in-frame AA deletion mutants exhibited defective phosphorylation: largely truncated or hypo-phosphorylation of both PER proteins compared to wt PER. A subset of these PER deletion mutations as well as PAS domain point mutations W448E in *mPer1* and W419E in *mPer2* were tested in a different human cell line (HEK293) through transient transfection using mammalian expression plasmids; transient co-expression of CK1δ produced much less phosphorylation of these mutant PERs than wt PER (Fig. S2). This is exciting but counterintuitive because PAS has not previously been directly implicated in PER phosphorylation or interaction with CK1.

**Figure 1:**
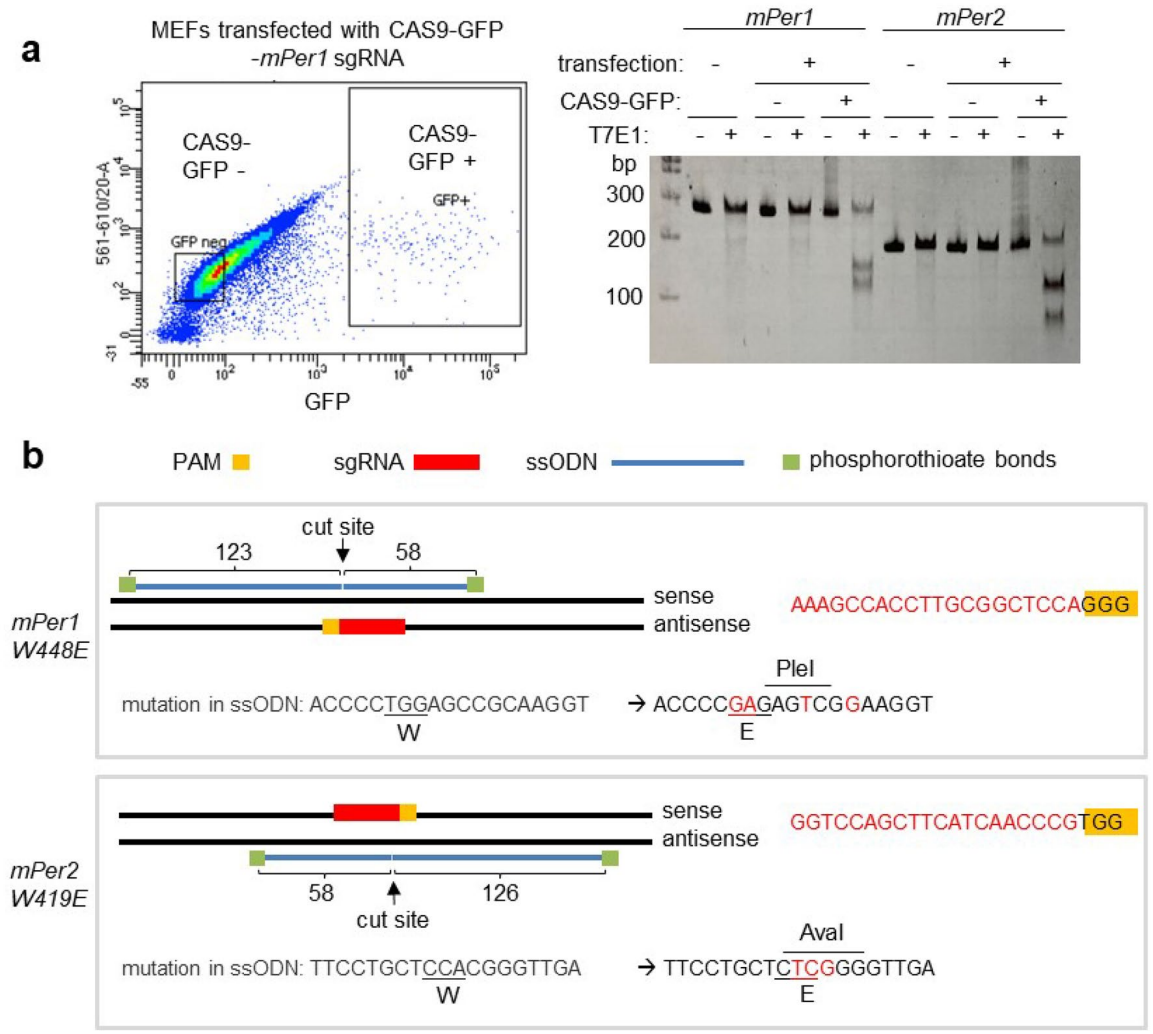
Strategy to knock-in mutations into *mPer1* and *mPer2* genes. (**a**) Efficient sgRNAs targeting *mPer1 W448* and *mPer2 W419* residues were selected by T7E1 assays in MEFs. Positive and negative transfected cells were selected by GFP expression and subjected to T7E1 assays. T7E1 results for less efficient sgRNAs are not shown. The original gel is presented in Fig. S8. (**b**) ssODNs for HDR are designed based on location of sgRNA. Note the asymmetric homologous arms.

**Figure 2:**
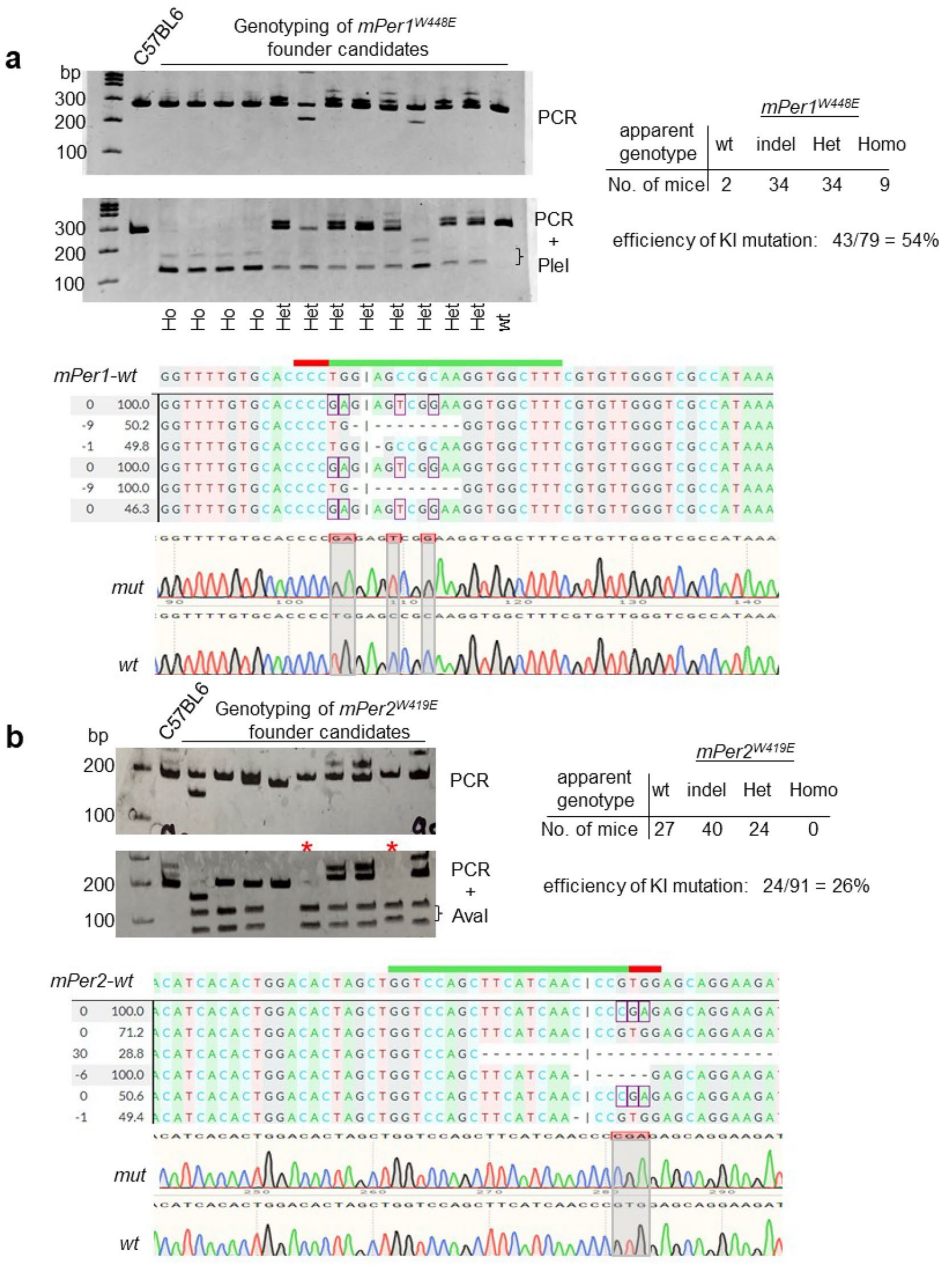
Knockin mutant mice are efficiently made by CRISPR-Cas9. (**a,b**) Genotyping by PCR and enzyme digestion showed efficient genome editing for two knockin mutations. Note that PCR amplicons indicated by a red asterisk in (**b**) were completely digested by AvaI. Some of the indel mutants along with the specific knockin mutants are shown by Sanger sequencing. The original gels are presented in Fig. S8.

All the data above strongly indicate that the PAS domain should be critical in circadian timing because PER phosphorylation is the basis of the mammalian circadian timer; thus mutations in and around the critical tryptophan residue and disrupting its phosphorylation should impair circadian rhythms. As discussed above, we aimed to generate tryptophan-to-glutamate mutations in *mPer1* and *mPer2,* respectively. We selected an efficient sgRNA sequence close to the residues by testing several sgRNA sequences around the residues. MEFs were transfected with all-in-one pAdTrack-Cas9-sgRNA plasmid followed by FACS sorting for positive cells (GFP from the all-in-one plasmid)^17^. These cells were subjected to T7E1 assays and the most effective ones were selected (Fig. 1a). When ssODNs were designed for homology-directed repair (HDR) to mutate W448E in *mPer1* and W419E in *mPer2*, novel restriction enzyme sites were added in the template to facilitate genotyping without sequencing PCR amplicons (Fig. 1b). These additional mutations are also necessary when digital PCR is designed to distinguish between wt and mutant alleles. Although annealing temperature is altered by single nucleotide polymorphisms, it is very challenging to develop an all-or-none annealing condition based on a single nucleotide mutation^53^. Cas9 protein along with sgRNA and ssODN were injected or electroporated into one-celled fertilized eggs to produce the two knockin mutant mice. A total of 990 embryo injections/electroporations (see “Materials and methods” section for a detailed breakdown) generated 79 and 91 live pups for *mPer1* and *mPer2* mutant mice, respectively. As summarized in Fig. 2a, we obtained 34 apparent heterozygous (het) and 9 apparent homozygous (ho) mutant mice for *mPer1*^*W448E*^ based on genotyping of tail tissues of founder mice (F0). Based on the total number of mice we examined and the number of mice harboring the mutant allele, KI efficiency was 54%. In addition, we obtained mice with 13 useful in-frame AA indel mutations which we expect to produce a more severe phenotype than the point mutations. For *mPer2*^*W419E*^, 24 apparent heterozygotes and no apparent homozygotes were produced (Fig. 2b). Although enzyme digestion of PCR amplicons suggested several homozygotes (see clones with red asterisk in Fig. 2b), they turned out to be all heterozygotes when assayed by digital PCR (see below). One of the two alleles had large deletions in these mice, which could not be amplified with the primer set and thus produced an unmixed sequencing chromatogram. It should be noted that low frequency of minor alleles due to mosaicism could be present in tail samples but not detected using conventional PCR-based genotyping method in F0 mice. When the mutant mice were genotyped by PCR, amplicon samples were run on both polyacrylamide gel (PAGE) and agarose gel to detect heteroduplex DNA and multiple on-target insertions (concatemers), respectively. It is known that heteroduplex DNA runs much larger than expected on PAGE in a sequence specific manner, and this heteroduplex mobility assay is widely used for genotyping^54,55^. We used this property for initial screening, but final genotype was confirmed by enzyme digestion and Sanger sequencing. As shown in Fig. 2 and Fig. S6, amplicons of larger than expected size are only visible in PAGE, but they are not detected on agarose gels, indicating that there are no multiple on-target insertions. This was subsequently confirmed by ddPCR (see “Genotyping for CRISPR-mutant mice can be streamlined by digital PCR” section results below).

Mosaicism can occur if Cas9-sgRNA continues to edit the genome after the one cell stage, and mosaicism in germline cells may be different from that in tail tissue. To confirm transmission of the mutations into the next generation and test mosaicism in the germline, we measured the genotypes of F1 mice using genomic DNA samples from ear tissue. After generating 2–3 F1 litters per founder, F1 genotypes were not different from those in tail tissue of F0 except in one instance. One of the *mPer1* mutant mouse lineages had a third allele in the F1 mice not seen in tail DNA from F0 mice. We did not detect third alleles in any of the F0 tail DNA samples, suggesting that any third alleles were not present or present at very low frequencies in the tail tissue. It thus appears that the rate of mosaicism in our procedures is very low overall, but not zero, warranting continued monitoring.

### Genotyping for CRISPR-mutant mice can be streamlined by digital PCR

From the conventional genotyping by PCR and enzyme digestion (Fig. 2), we believed 5 homozygotes for *mPer2*^*W419E*^ mutant mice were made because PCR amplicons from these mice were completely digested by AvaI. However, we did get only 50% instead of 100% heterozygous pups from breeding between these mutant and wt mice suggesting that one allele in these mice had large indels and thus the small PCR amplicon using the primer set could not be generated from these alleles. Indeed, PCR producing a large amplicon, ~ 1.2 kb revealed large deletion alleles in three of the mice (Fig. S3a). Despite several attempts using primers generating ~ 3 kb amplicons, we were not able to produce mutant PCR amplicons from the remaining two mice. Since large indels are not rare events and correct genotyping is critical when selecting founder mice, we developed a droplet digital PCR (ddPCR) assay for genotyping, which can quantify the number of the mutant copy over the copy number for a reference gene in the same sample in a PCR reaction and thus reveal correct genotypes regardless of indel size and digestion pattern. If a mutant probe is used, the ratio would be 1, 0.5 and 0 for homozygotes, heterozygotes and wt mice, respectively (Fig. S3b). For F0 mice, this genotyping approach would still not be definitive because of the possibility of mosaicism.

**Figure 3:**
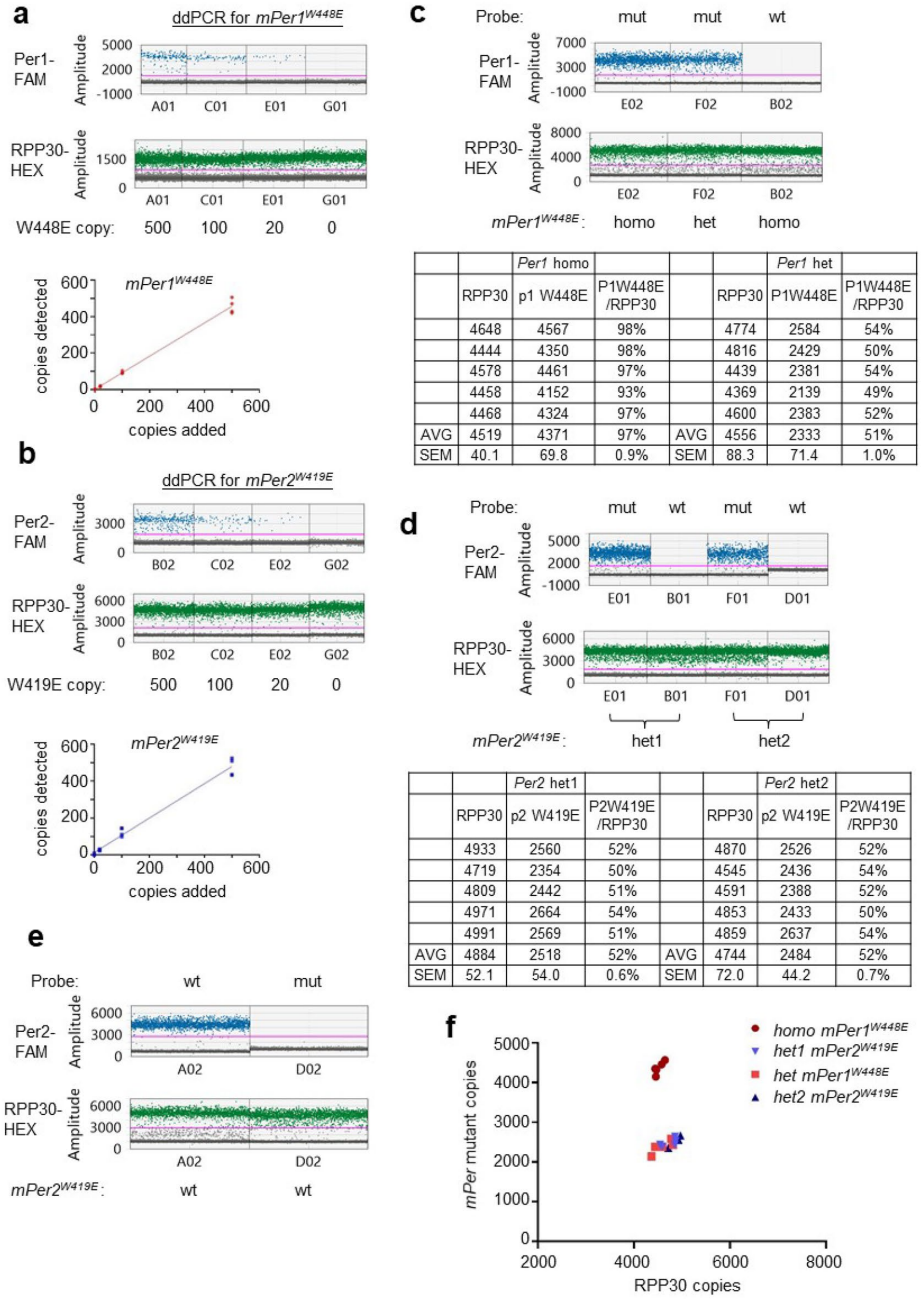
Digital PCR can be used to genotype CRISPR mutant mice that cannot be genotyped by PCR analysis. (**a,b**) ddPCR was optimized using genomic DNA obtained from the mutant mice in Fig. 2. Our ddPCR conditions can reliably detect as low as 8 mutant copies in a reaction when they are mixed with 5000 copies of wt allele (Fig. S4). N = 4 each. (**c,d**) ddPCR produced reliable genotyping results even in compound heterozygotes which could not be genotyped by conventional PCR and enzyme digestion. PCR amplicons could not be generated from one of two alleles in two compound heterozygotes in (**d**) because they presumably had very large deletions. Note that the wt probe could not detect wt allele in these heterozygotes. N = 5 each. (**e**) The wt probe can detect wt allele. (**f**) Digital PCR can be a streamlined procedure for rapid genotyping of CRISPR mutant mice even for mutant mice with large indels.

Sensitivity and accuracy of ddPCR were assessed by generating standard curves for low copy numbers of the mutant genomic alleles spiked into 5,000 copies of the wt genomic allele (Fig. 3a,b, Suppl Fig. S4). When 20, 100 and 500 copies of the mutant alleles were spiked into 5,000 copies of the wt allele, the ddPCR produced a strong linear correlation between added and detected copies (Fig. 3a,b) (R^2^ > 0.98). The reference probe for *RPP30* gene also consistently detected ~ 5000 copies in these samples. Genotyping of both *mPer* mutant mice using this ddPCR protocol produced reliable results which were consistent with those obtained by enzyme digestion of PCR amplicons, except for five *mPer2*^*W419E*^ mutant mice (Fig. 3c–f). Three of the *mPer2* mutant mice were compound heterozygotes, which matches the results of PCR genotyping producing a larger amplicon (Fig. S3a). The other two compound heterozygous *mPer2*^*W419E*^ mutant mice with putative large deletions could be also confirmed by ddPCR (Fig. 3d,f). ddPCR with the mutant probe on these samples produced ½ signals relative to those of the RPP30 probe whereas the wt probe did not produce any signal. These results demonstrate that the conventional PCR-based genotyping may not be suitable for all CRISPR mutant mice, especially ones with large indels, for which ddPCR is a much more tractable approach.

Because off-target insertions of the repair templates may affect expression of the inserted genes or produce small pieces of PER proteins, they could affect circadian mechanisms. To detect off-target insertions, ddPCR was performed using primers binding inside of the repair template. When copy numbers were compared between in-in and in–out primers, both ddPCR pairs produced similar copy numbers (Fig. S5) indicating that there were no off-target insertions in these mice.

### Targeted mutations by CRISPR-Cas9 are significantly less efficient in MEFs compared to mice

Although CRISPR genome editing can be done in vivo in a much more efficient manner compared to the conventional knockin methods, it still requires a lot more resources including space for animals compared to cell-based systems. As with many biological fields, circadian mechanisms can be studied in cells because the circadian clock is cell autonomous. Although demand for genome editing in cells in the circadian field is increasing because molecular mechanisms can be better studied in cell models such as MEFs and U2OS, there are only few cases where specific mutations other than knockouts in clock genes have been made in these cells. Many clock mutant mice are readily available, but the polymorphisms in clock genes in the human gene pool are orders of magnitude larger. Implementing a streamlined workflow for specific genome editing such as introducing SNPs or short mutations in MEFs derived from existing mutant mice would greatly facilitate mechanistic studies of diverse genetic conditions. The approach will be also valuable in other fields where non-transformed cell models such as MEFs are required.

**Figure 4:**
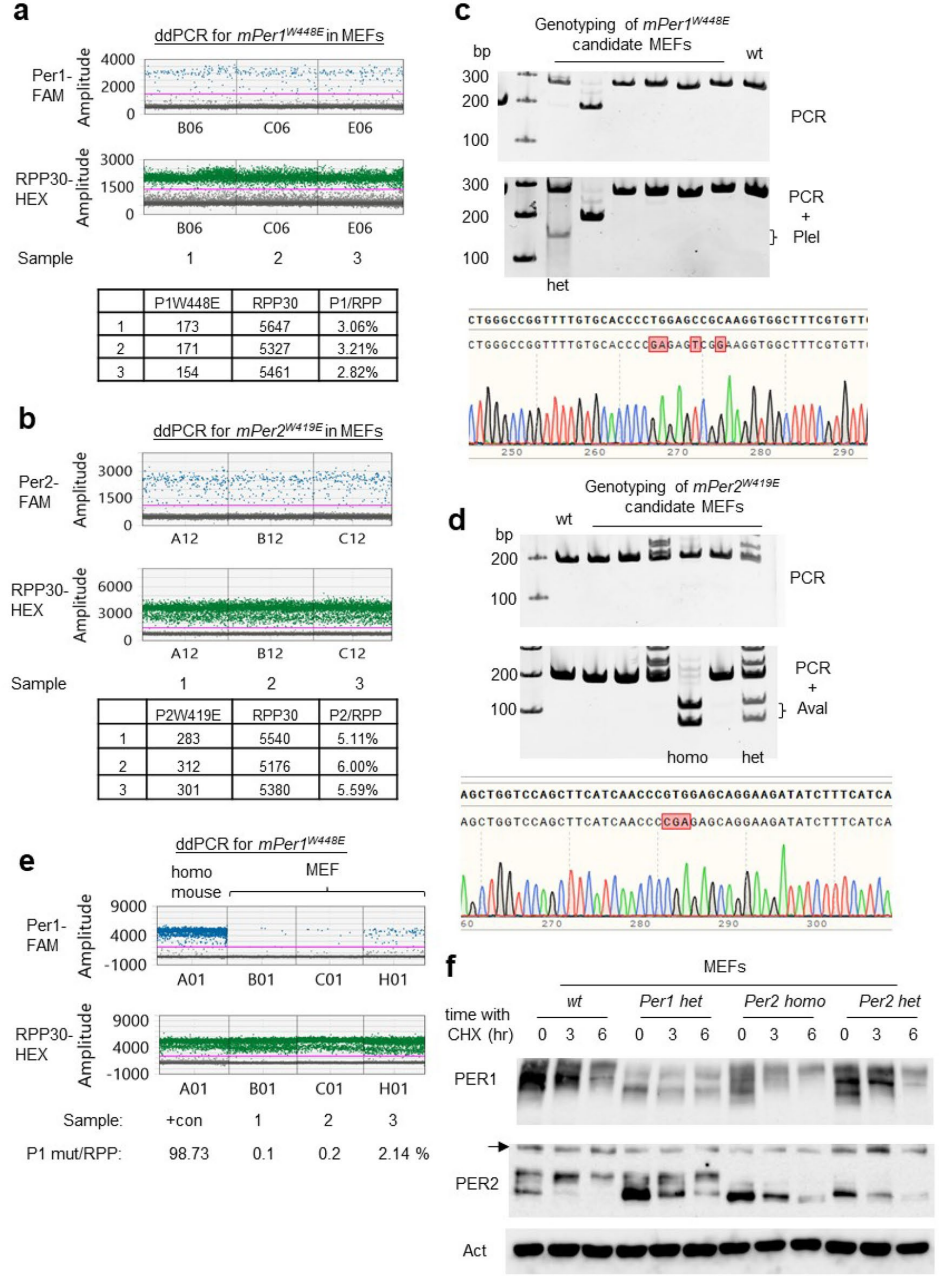
Digital PCR can be used for accurate quantification of specific genome editing in MEFs. (**a,b**) Frequency of two *mPer* mutations were quantified by ddPCR in MEFs transfected with the CRISPR reagents. ddPCR was performed on three independent samples each. (**c,d**) *mPer* mutant clones were successfully isolated from screening of 15 random clones each. Genomic DNA from these clones were amplified by PCR and digested with PleI (*mPer1*) and AvaI (*mPer2*). The original gels are presented in Fig. S8. (**e**) Digital PCR can be a powerful tool to optimize CRISPR-HDR protocols. Varying amounts of *mPer1*-HDR template and all-in-one Cas9-sgRNA plasmid were co-transfected into MEFs followed by FACS sorting. Genomic DNA from a homozygous *mPer1* mutant mouse was used as a positive control. (**f**) PAS mutant mPER proteins show defective phosphorylation. Although both PER1 and PER2 mutants showed defective phosphorylation, *mPer2*^*W419E*^ mutant is apparently more defective in phosphorylation. The original blots are presented in Fig. S7.

To streamline specific genome editing in MEFs, we assessed the efficiency of generating the *mPer1*^*W448E*^ and *mPer2*^*W19E*^ mutations using the same sgRNA and repair templates in MEFs that we had used for CRISPR editing of mouse zygotes. To compare the efficiency in a quantitative manner and assess the downstream clonal isolation workload, ddPCR was performed on FACS-sorted positive cells after transfection of the reagents as described in Fig. 1a. In bulk MEFs transfected with the same sgRNA and ssODN for *mPer1*^*W448E*^ mutation, ~ 3% of wt *mPer1* were converted into the mutant allele (Fig. 4a). The efficiency was 5–6% for *mPer2*^*W419E*^ (Fig. 4b). Although these numbers are significantly lower than those in mice, it is still very reasonable for researchers to successfully isolate desirable mutant clones by screening only a few dozen random clones using simple molecular techniques such as enzyme digestion of PCR amplicons as described above. To verify this efficiency in isolated MEF clones, some of the bulk positive cells above were singly sorted into 96 well plates and expanded for the PCR analysis followed by enzyme digestion. When we screened 15 clones each, 1 heterozygote clone for *mPer1*^*W448E*^ and 1 heterozygote clone and 1 homozygote clone for *mPer2*^*W419E*^ were isolated, roughly matching the ddPCR numbers (Fig. 4c,d). The ddPCR assay is also a powerful tool when CRISPR-HDR protocols are being optimized. Efficiency of HDR was greatly affected by the ratio of CRISPR plasmid to donor template in co-transfection (Fig. 4e).

When PER proteins were probed in these mutant MEF clones, both PER proteins were dramatically less phosphorylated compared to control MEFs (Fig. 4f). Existing PER proteins are progressively phosphorylated when do novo translation is inhibited by cycloheximide (CHX). These data along with the data in Fig. S2 strongly suggest that PAS dimerization is critical for PER phosphorylation and probably for the clockwork as well. Because MEFs are not transformed cancer cells and thus are frequently used as an in vivo model, our data strongly support that MEFs can be an effective platform to study in vivo cell physiology, and this is even more compelling by already available numerous genetic mouse models.

## Discussion

**Figure 5:**
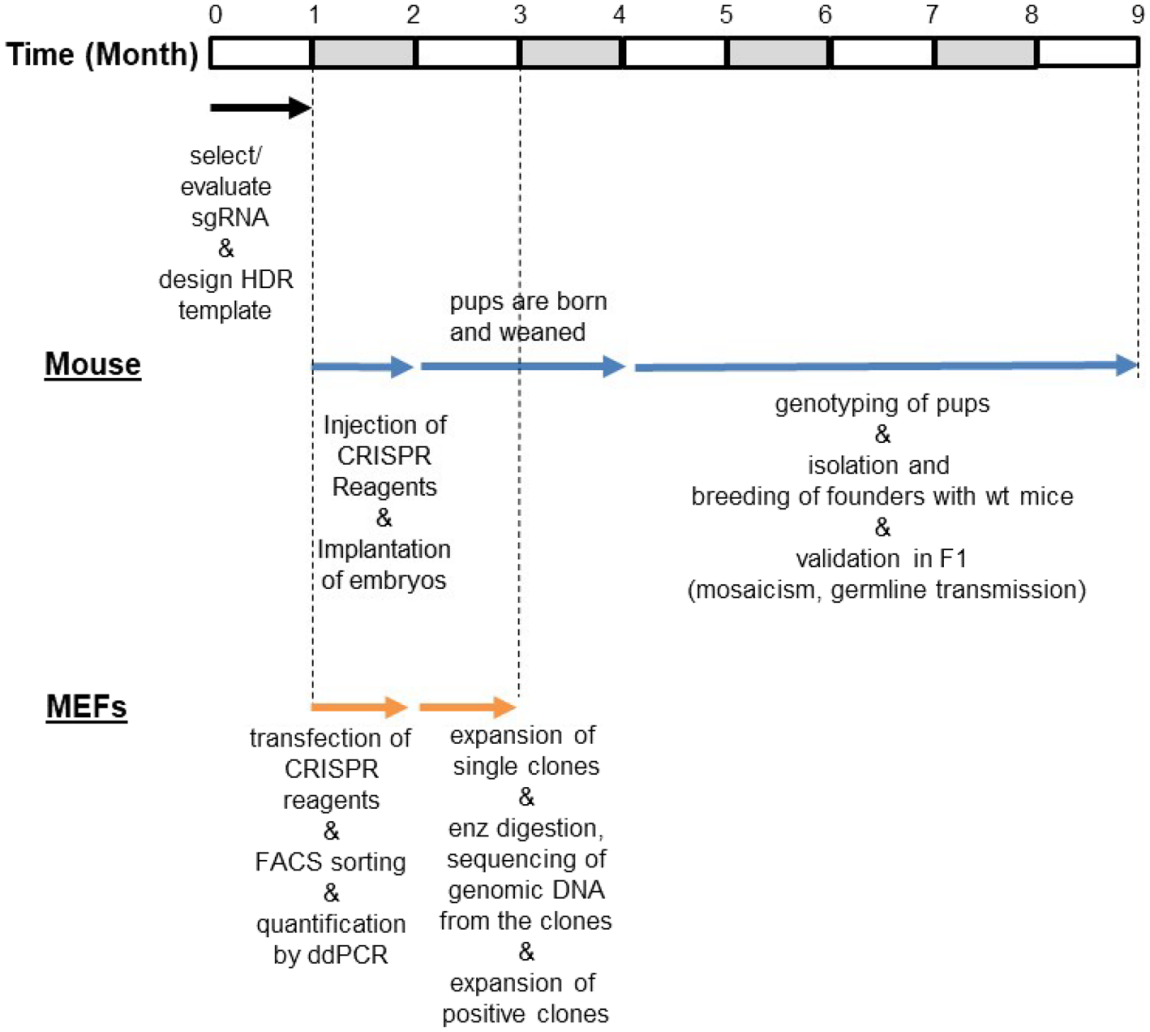
Timeline comparison in CRISPR-knockin mutation between mice and MEFs. Reasonable amounts of time for troubleshooting and optimization typical for a small laboratory are included in this timeline. Steps from injection of CRISPR reagents into fertilized eggs to producing pups are usually done by a dedicated animal facility. The rest of the animal work and all the work in MEFs can be done by an individual laboratory.

As with most biological fields, our current understanding of the circadian clock in mammals has been largely established by reverse genetics in mice that have led to identification of a dozen essential clock genes to date^56^. Although the mechanistic insights from these mutant mouse models cannot be overstated, the traditional mutant mouse models based on gene targeting by homologous recombination required tremendous resources and a long time (18–24 months) even to assess if desired mutant models were made. In that sense, CRISPR revolutionized how mutant animal models are generated. It is faster (~ 9 months) and much more affordable and efficient (Fig. 5). In addition, as in our work reported here, specific genome editing by CRISPR generated useful by-products such as knockouts and AA indels. Because these AA indels would disrupt the function of the motif more severely than the single AA mutation in the motif—and the larger the indel, the more severe the phenotype may be—these indel mutants along with the specific mutant animals would provide more complete or novel insights into the function of the motif and the whole protein. As in our study, compound heterozygotes would be the most predominant genotype with successful injection of CRISPR reagents and sgRNA efficiency. ddPCR assays are very useful for accurate genotyping of these compound heterozygotes, especially alleles with large indels. Although our ddPCR results suggest that there are no off-target KIs, there could be off-target indel mutations in our mice and MEFs, and this could affect circadian mechanisms. However, since homologous mutations were generated in two redundant genes using different gRNA, and phenotypes can be compared between the two mutants, off-target effects could be detected if there are any. New genome editing techniques such as Prime editing with pegRNA can dramatically decrease the likelihood of off-target mutations^57^.

Cell culture models will continue to be an effective platform for studying molecular mechanisms in many biological pathways including circadian biology, given their time- and cost-efficiencies. Although the genome in cell culture models can be precisely edited using CRISPR-Cas9, it is also important to recognize such genome edited cell models are very rare in general. This may reflect that precise genome editing is still not practical in many small laboratories because HDR frequency is significantly lower than that of NHEJ, and thus it is challenging to isolate HDR clones out of much more abundant NHEJ mutant clones. Frequency of the specific mutation could be easily estimated by enzyme digestion followed by gel electrophoresis if a novel digestion site is added into the repair template, and the HDR frequency is high enough. However, we could not detect digested fragments from PCR amplicons prepared from genomic DNA of our bulk sorted MEFs on agarose gels. When HDR efficiency is 3–6% as in our bulk sorted cells, we believe the densitometric method is not sensitive enough to detect digested fragments on agarose gels. Next-generation sequencing (NGS) would be the gold standard for accurate quantification of HDR, but it is not practical especially when many mutant cell lines are generated, or HDR conditions are not yet optimized. Downstream work flow and effort would be greatly affected by the efficiency of HDR. For example, if the efficiency is ~ 0.1%, at least several hundreds of random clones need to be expanded and interrogated by PCR analysis and sequencing, which would not be practical. Digital PCR assays can be a powerful tool when a specific mutant cell clone needs to be isolated, because frequency of the mutation can be accurately measured from a heterogenous population of mutant clones, which will allow researchers to estimate the number of single clones to be analyzed to isolate a few desirable mutant clones or to optimize the conditions to increase HDR efficiency (Fig. 5).

As our understanding of many important biological pathways is becoming mature by identification of most of the essential genes, a next frontier in biology would be to learn how polymorphisms in these genes affect the pathways and physiology. For circadian biology, it has been already demonstrated that some of these polymorphisms are associated with circadian disorders such as Familial Advanced Sleep Phase Syndrome^58,59^. We believe our CRISPR method combined with ddPCR can be a streamlined process to generate any simple mutant KIs in MEFs to study human genetics, if relevant human physiology can be studied in MEFs like circadian biology.

Although we cannot generalize CRISPR efficiency for HDR in mice, available data suggest it is close to 100% if the sgRNA is properly selected, and reagents are successfully delivered. Considering this high efficiency, the short time frame from CRISPR design to obtaining founder mice, and the potential to generate combined mutations by breeding, it may be more practical to generate mutant mice rather than individual mutant cell clones in some cases. Mouse models are obviously more versatile since whole animal physiology can be studied in single and combined mutant mice as well as molecular mechanisms in diverse cells or tissues isolated from these mutant mice.

In summary, when the same sgRNA and HDR-template are used, HDR efficiency is dramatically higher in fertilized eggs compared to MEFs, and digital PCR is an extremely powerful tool for genotyping of these CRISPR mutant mice and for quantitative analysis of the mutation frequency if the genome editing is done in cells. Using our protocols, we believe that any simple mutant model can be efficiently generated in MEFs or other CRISPR-compatible cell lines, even in small laboratories, because implementation of ddPCR is fairly straightforward. The cost of the ddPCR equipment is nontrivial, but such instruments are becoming increasingly available. Thus, our cell-based methodology may facilitate faster and higher throughput studies of genetic polymorphisms in the circadian system and in other areas of biology.

## Materials and methods

### Experiments in U2OS and HEK293 cell lines

#### Isolation of mutant *Per1* and *Per2* clones in U2OS cells

 ~ 30 clones from the above FACS-sorted cells were expanded and subjected to immunoblotting for PER to select in-frame mutant clones. Positive clones were identified by aberrant mobility of PER and confirmed by TA cloning followed by Sanger sequencing. The results are shown below.

**Table:** 

wtPER1: GEYVTMDTSWAGFVHPWSRKVAFVLGRHKVRTAPLNEDVFTPPAPSPAPSLDTD
#7 Allele1: GEYVTMDTSWAGFVHP-----------------------------SPAPSLDTD Allele2: frameshift mutation
#16 Allele1: GEYVTMDTSWAGFV----------LGRHKVRTAPLNEDVFTPPAPSPAPSLDTD Allele2: frameshift mutation
wtPER2: RARNGEYITLDTSWSSFINPWSRKISFIIGRHKVRVGPLNEDVF
#2 Allele1: RARNGEYITLD--------------SFIIGRHKVRVGPLNEDVF Allele2: frameshift mutation
#5 Allele1: RARNGEYITLDTSWSSF--PWSRKISFIIGRHKVRVGPLNEDVF Allele2: frameshift mutation
#8 Allele1: RARNGEYITLDTSWSSFIN---RKISFIIGRHKVRVGPLNEDVF Allele2: frameshift mutation

### Experiments in mice and MEFs

#### Generation of mutant mice and genotyping

All mice were maintained in a climate-controlled room and used according to the Florida State University Animal Use Committee’s guidelines. All experiments involving animals were performed according to approved protocols by FSU ACUC, protocol number: 202200000021. All methods are reported in accordance with ARRIVE guidelines. We used about equal numbers of male and female mice.

Injection of ribonucleoprotein complex and ssODNs into one-day fertilized eggs, and generation of pups from pseudopregnant mice were done in the C57BL/6J strain at the UT Southwestern Medical Center core facility according to approved protocols by UTSW ACUC. Briefly, crRNA (IDT, Inc.) and tracrRNA were annealed and mixed with Cas9 protein to form a ribonucleotide protein complex. The ssODN (IDT, Inc.) was added to the mix and the cocktail was microinjected into the cytoplasm of fertilized one-cell eggs isolated from superovulated females. The eggs were incubated in media containing cytochalasin-B immediately before and during microinjection to improve egg survival. Alternatively, CRISPR reagents were delivered to the cytoplasm via electroporation using the Gene Pulser (BioRad, Hercules, CA, USA). The surviving eggs were transferred into the oviducts of day 0.5 pseudopregnant recipient ICR females (Envigo, Inc.) to produce putative founder mice. See below for breakdowns of CRISPR delivery methods and results.

Founder mice (F0) were identified via PCR using the primers described below. Tail tissues from F0 mice were obtained when these mice were weaned at 3 weeks old. PCR amplicons were run on both polyacrylamide gels (PAGE) and agarose gels, digested with PleI (NEB Inc.) for *mPer1*genotyping and AvaI (NEB Inc.) for *mPer2* genotyping and sequenced. In the majority of the clones, two different Sanger sequencing traces were mixed due to different indels and/or the HDR mutation in two alleles. These results were deconvoluted by a computer algorithm called DECODR v3 (https://decodr.org/) into two separate traces. Accuracy of the deconvolution was confirmed by TA cloning if the decoding results were ambiguous (see DECODR vs TA cloning in Supplementary uncropped images and raw data). TA cloning was performed according to the manufacturer’s protocol (ThermoFisher K4575) and more than 8 clones per sample were sequenced.

##### Guide RNA

gRNA for *mPer1*: aaagccaccttgcggctccagRNA for *mPer2*: ggtccagcttcatcaacccg.

##### PCR primers for T7E1 and genotyping

**Table:** 

Assay	Gene	Primers		Size of amplicon
Genotyping	*mPer1*	CCCATTCACTGATACCCACTTT	AAGGAGAACTCAGTCCTTTCCC	275
Genotyping	*mPer2*	TTCGATTATTCTCCCATTCGAT	GAGAGGTGAGAATAGGCCAAAA	193
Genotyping-large	*mPer2*	GATCTGATCGAGACGCCTGTG	ATGGCTGCAACACAGACGAT	1156
T7E1	*mPer1*	CCCATTCACTGATACCCACTTT	AAGGAGAACTCAGTCCTTTCCC	275
T7E1	*mPer2*	TTCGATTATTCTCCCATTCGAT	GAGAGGTGAGAATAGGCCAAAA	193

For Fig. S3a, the genotyping-large primers were used to generate a large amplicon, ~ 1.2 kb.

##### ssODN sequence

*mPer1*: CctgcgcccacagTACTGCAGCTGGCAGGCCAGCCCTTTGACCATTCCCCTATTCGCTTCTGTGCTCGGAACGGGGAATATGTCACCATGGACACCAGCTGGGCCGGTTTTGTGCACCCC gaGAGtCGgAAGGTGGCTTT CGTGTTGGGTCGCCATAAAGTGCGCACgtaagggaactgtg.*mPer2*: ttgccccctgctgtgagaggtgagaataggccaaaatcccccaaaacccacagagtggaaccctgggagcactcacACCCTGACTTTGTGCCTCCCAATGATGAAAGATATCTTCCTGCTCtcgGGGTTGATGAAGCTGGACCAGCTAGTGTCCAGTGTGATGTACTCCCCGTTGCGGGTGCGG.

##### Methods for delivery of CRISPR reagents and outcomes

**Table:** 

Construct	Embryos electroporated	Embryos transferred	Females transferred	Females pregnant	Litter at birth	Pups surviving
BioRad electroporations						
Per1	369	369	11	11	72	62
Per2	184	170	5	4	33	28
Cytoplasm injections						
Per1	102	93	3	2	18	17
Per2	335	304	9	8	78	63

Method	Cas9 Protein (IDT) (ng/ul)	sgRNA (ng/ul)	Repair template (ng/ul)			
Reagent concentrations						
Electroporations	400	300	400			
Cytoplasm Injections	50	50	50			

Founder mice were tested for mosaicism and germline transmission by measuring and comparing alleles between F0 and F1 born from mating between F0 and wt mice. For all F0 mice used for breeding, there was 100% germline transmission and one identified mosaic mouse.

**Table:** 

*mPer1*			F0 x wt								
F0-tail			F1-I			F1-II					
ID	Allele 1	Allele 2	Allele 1	Allele 2	n	Allele 1	Allele 2	n			
6	W448E	W448E	W448E	WT	4						
7	W448E	W448E	W448E	WT	8						
12	W448E	W448E	W448E	WT	2						
13	W448E	60 del	W448E	WT	2	60 del	WT	3			
16	14 del	72 del	14 del	WT	4	72 del	WT	5			
27	W448E	W448E	W448E	WT	7						
34	W448E	14 del	W448E	WT	4	14 del	WT	4			
41	3 del	Large del	3 del	WT	5	Large del	WT	2			
35	9 del	1 del	9 del	WT	4	1 del	WT	2			
71	W448E	W448E	W448E	WT	5						

Mosaic mouse			F1-I			F1-II			F1-III		
53	3 del	WT	3 del	WT	5	WT	WT	4	W448E	WT	3

**Table:** 

*mPer2*			F0 x wt					
F0-tail			F1-I			F1-II		
ID	Allele 1	Allele 2	Allele 1	Allele 2	n	Allele 1	Allele 2	n
94	190 del	18 del	190 del	WT	4	18 del	WT	2
98	6 del	Large del	6 del	WT	2	Large del	WT	6
97	W419E	WT	W419E	WT	5	WT	WT	3
100	W419E	21del (413–419)	W419E	WT	6	21del (413–419)	WT	2
103	W419E	Large del	W419E	WT	4	Large del	WT	4
106	W419E	Large del	W419E	WT	2	Large del	WT	6
136	W419E	WT	W419E	WT	4	WT	WT	1
162	W419E	30 del	W419E	WT	1			

#### T7E1 assays and isolation of HDR mutant clones in *mPer2*^*Luc*^ MEFs

For transfection in MEFs, cells were prepared as described above. 900 ng of all-in-one plasmids were used for Fig. 1, and 900 ng all-in-one plasmids + 300 ng repair templates were used for HDR mutations. For Fig. 4e, 300 + 900 (1), 600 + 600 (2) and 900 + 300 (3) ng DNA were used for varying amounts of all-in-one plasmid to repair template, respectively. Single clones were identified by immunoblotting and sequencing as described above.

### Experiments in droplet digital PCR (ddPCR)

Droplet digital PCR reagents were purchased from Bio-Rad and reactions were set up as follows. Relevant info according to the 2020 Minimum Information for Publication of Quantitative Digital PCR Experiments (MIQE) guidelines^60^ is reported in the manuscript and summarized in Table 7 in Supplementary uncropped images and raw data.

**Table:** 

Component	Volume per reaction, μl	Final concentration
2× ddPCR Supermix for Probes (No dUTP) 186–3023	12.5	1×
20× Per HDR Assay (FAM)	1.5	900 nM (primer)/250 nM(probe)
20× RPP30 Assay (HEX)	1.5	900 nM (primer)/250 nM (probe)
HindIII	0.6	–
Genomic DNA	1 (14.25 ng)*	5000 copies/μl
RNase/DNASE-free water	7.9	–
Total volume	25	–

*14.25 ng is equal to ~ 5000 copies of a mouse allele. gDNA was dissolved in deionized water and aliquoted and stored at − 80 °C after concentration was measured by Nanodrop.

The following primers and probes were used.ddPCR assay primers:

**Table:** 

Genes	Forward primers	Reverse primers	Products (bp)
*mPer1* (in–out)	TGACCATTCCCCTATTCGCTT	CCATGCCATGTCCATACCAC	186
*mPer1* (in-in)	CCCCTATTCGCTTCTGTGCT	TTACGTGCGCACTTTATGGC	125
*mPer2* (in–out)	TTCGATTATTCTCCCATTCGAT	GAGAGGTGAGAATAGGCCAAAA	193
*mPer2* (in-in)	CTCCCATTCGATTCCGCACC	GAGCACTCACACCCTGACTT	131
*mRPP30*	AAGAAACCACGGCCATCAGAAG	GGGTTTTATTTGCTGTTTTAATGGTC	231

Before selecting final probes, specificity and efficiency of several candidate probes were tested.ddPCR probes:

- *mPer1_W448* ddPCR wild-type probe: ACCCCTGGAGCCGCAAGGT (/56-FAM/ACCCCTGGA/ZEN/GCCGCAAGGT/3IABkFQ/).
- *mPer1_W448E* ddPCR mutant probe: ACCCCGAGAGTCGGAAGGT (/56-FAM/ACCCCGAGA/ZEN/GTCGGAAGGT/3IABkFQ/).
- *mPer2_W419* ddPCR wild-type probe: TTCCTGCTCCACGGGTTGA (/56-FAM/TTCCTGCTC/ZEN/CACGGGTTGA/3IABkFQ/).
- *mPer2_W419E* ddPCR mutant probe: AGCTTCATCAACCCCGAGAG (/56-FAM/AGCTTCATC/ZEN/AACCCCGAGAG/3IABkFQ/).
- *mRpp30* reference probe: CTGCCTCCTCCCCTTCGTAG (/5HEX/CTGCCTCCT/ZEN/CCCCTTCGTAG/3IABkFQ/).

Droplets were generated from 20 μl of the samples and subjected to thermal cycles as follows. Annealing temperature was tested at 55–65 °C to optimize clear droplet separation.

**Table:** 

Cycling Step	Temperature °C	Time	Ramp Rate	# of cycles
Enzyme Activation	95	10 min	2 °C/s	1
Denaturation	94	30 s		40
Annealing/Extension	55	1 min		40
Enzyme Deactivation	98	10 min		1
Hold (optional)	4	Infinite		1

After the PCR amplification, the plate was transferred into the Bio-Rad QX-100 Droplet Reader. All assays were analyzed using the QX200 droplet reader and Quantasoft analysis pro (Bio-Rad).

Average number of droplets was about 16,000 partitions in a total volume of 20 μl. It corresponds to a droplet volume of 1.25 nl.

#### LoB and LoD of ddPCR assays

An average false positive rate (A_FP_) was measured from 38 negative samples. A total of 24 false-positive droplets were detected in 38 reactions (A_FP_ = 0.71). 97.4% of the reactions (37/38) contained < 4 false positive droplets, while 89.5% of the reactions (33/37) contained < 3 false positive droplets. The LoB was calculated and set at 3 to maintain an α error lower than 5%. α error indicates that the chance of detected false positive droplet counts in a negative control sample is greater than LoB. The LoD was calculated and set at 8 to maintain a β error lower than 5%. β error indicates that the probability of detected droplet counts is lower than LoB when a sample is prepared at the LoD^61^.

## Supplementary Information

Supplementary Information.

Supplementary Figures.

## Data Availability

All data generated or analysed during this study are included in this published article and its Supplementary Information files.
