# Supplementary material for: Generation of CRISPR-Cas9-mediated knockin mutant models in mice and MEFs for studies of polymorphism in clock genes
Source: Sci Rep. 2023 May 19;13:8109. doi: 10.1038/s41598-023-35203-7 (PMC10198968; doi:10.1038/s41598-023-35203-7)
Supplement: Supplementary file 2 — Supplementary Figures. [file 41598_2023_35203_MOESM2_ESM.pdf]

Supplementary Information for

Generation of CRISPR-Cas9-mediated knockin mutant models in mice and MEFs for studies of polymorphism in clock genes

Authors and Affiliations:

Kwangjun Lee[1] and Choogon Lee[1,*]

[1]Department of Biomedical Sciences
Program in Neuroscience
College of Medicine
Florida State University
1115 West Call Street
Tallahassee, FL 32306, USA

This PDF file includes:

Figs. S1 to S8

Fig S1

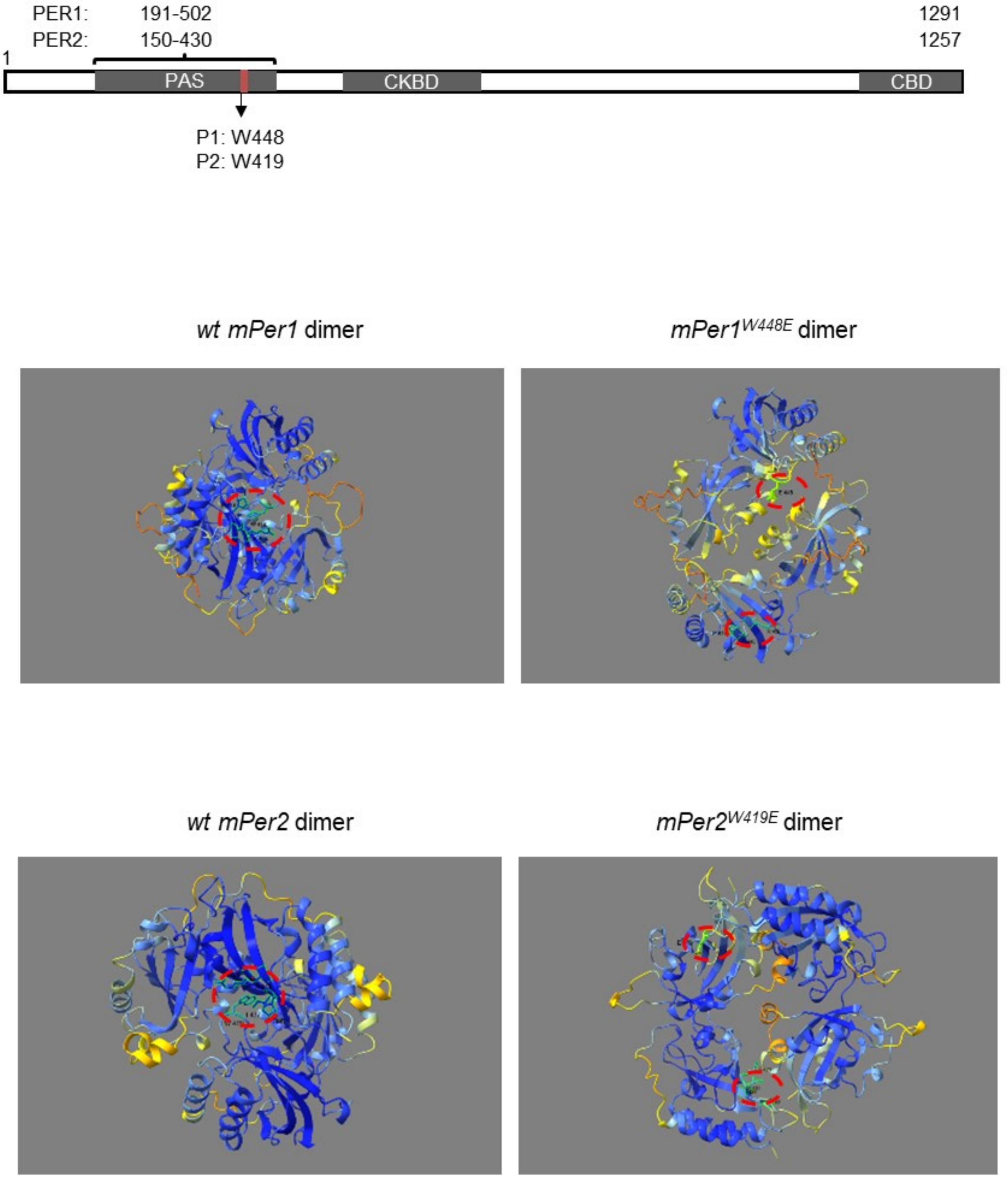

Fig S1. Mutations *mPer1^{W448E}* and *mPer2^{W419E}* are disruptive SNPs in mPER1 and mPER2 homodimerization. When the mutations were introduced into PAS dimers and simulated by the AlphaFold program, the hydrophobic bonding indicated by red circles was completely disrupted. Mutant PAS homodimers are simulated based on the published wt PAS dimer structure.

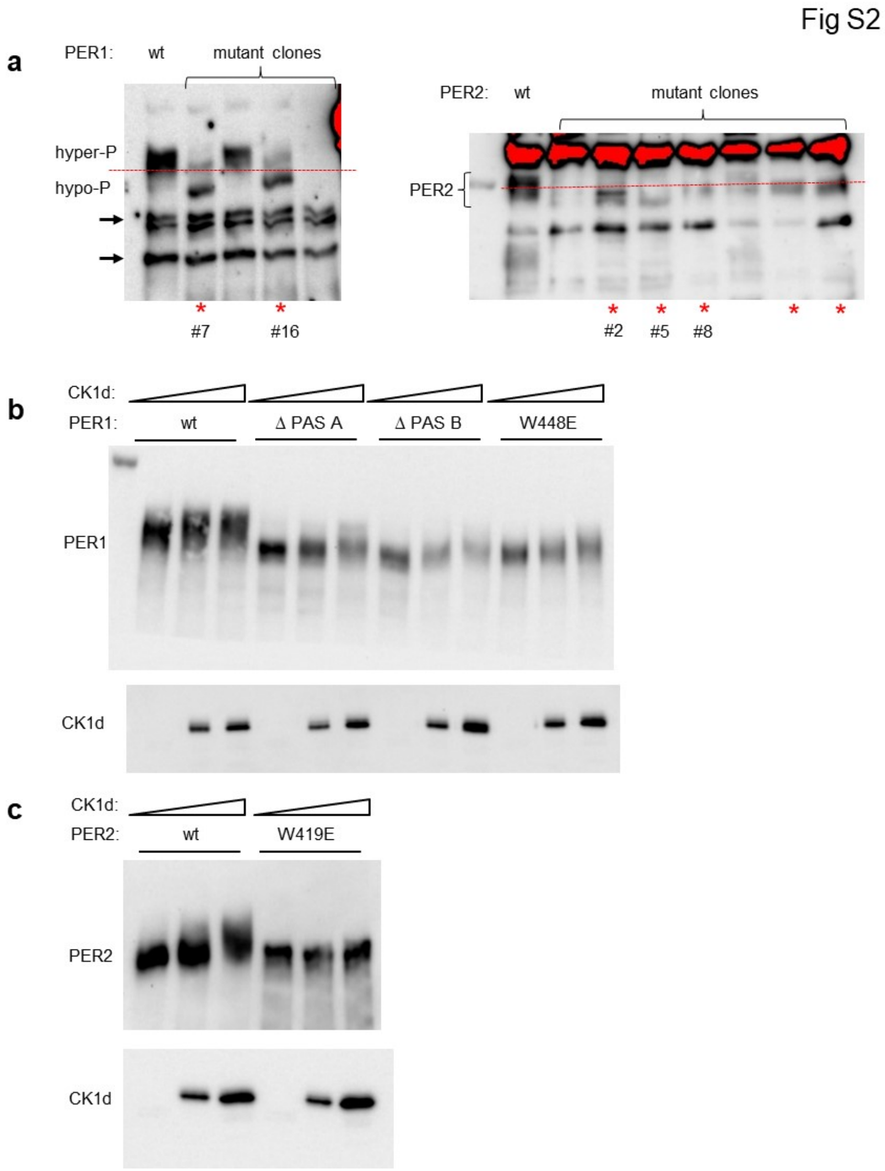

Fig S2. Random AA deletions in PAS B domain induce defective phosphorylation in U2OS cells. (a) hPER1 W448 and hPER2 W421 (conserved residues in human PER proteins) were targeted by CRISPR without HDR templates in U2OS cells. Note that PER proteins in samples indicated by red asterisks show all hypophosphorylated PER compared to wt PER. The mutant *hPer* genes from the numbered clones were sequenced (see the Materials and Methods). These clones were selected from screening 30 single clones each. Arrows indicate nonspecific bands. **(b, c)** When mutant mPER proteins with defective PAS domains were transiently co-expressed with CK1δ in HEK293 cells, they were all hypophosphorylated similar to the mutant endogenous hPER in U2OS cells. Δ PAS A and Δ PAS B represent mutant PER1 with deletions of AA 244-252 in PAS A and AA 444-448 in PAS B domain, respectively. Three different amounts of CK1δ were used: 0, 5 and 25 ng. *mPer* was fixed at 300 ng. The original blots are presented in Fig S7.

Fig S3

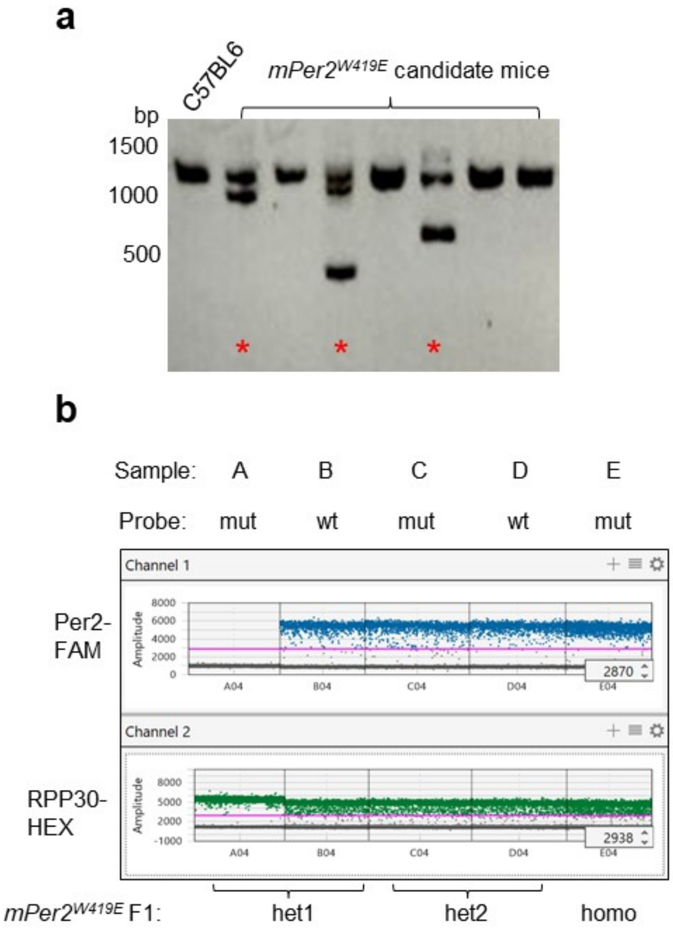

F1 samples	Mouse ID	probe	ref	RPP30	probe	P2W419 /RPP30
A	P2 W419 #400 large del/+	w419E probe	RPP30	4720	0	0.0%
B	P2 W419 #400 large del/+	wt probe	RPP30	4832	2326	48.1%
C	P2 W419 #232 W419E/+	w419E probe	RPP30	4732	2277	48.1%
D	P2 W419 #232 W419E/+	wt probe	RPP30	4811	2345	48.7%
E	P2 W419 #402 W419E/W419E	w419E probe	RPP30	4945	4865	98.4%

Fig S3. Digital PCR can be used to genotype CRISPR mutant mice with large indel alleles which cannot be analyzed by conventional PCR. (a) Increased PCR amplicon size can detect some large indel alleles. The amplicons indicated by red asterisks show that deletions in one allele in these mutant mice are larger than the amplicon size in Fig 2. The original gel is presented in Supplementary Excel File.

(b) Digital PCR genotyping of F1 pups between wt and apparent F0 *mPer2* homozygotes (A-D) and two apparent F0 heterozygotes (E) revealed that the conventional PCR analysis produced incorrect genotyping results of the founder mice.

Fig S4

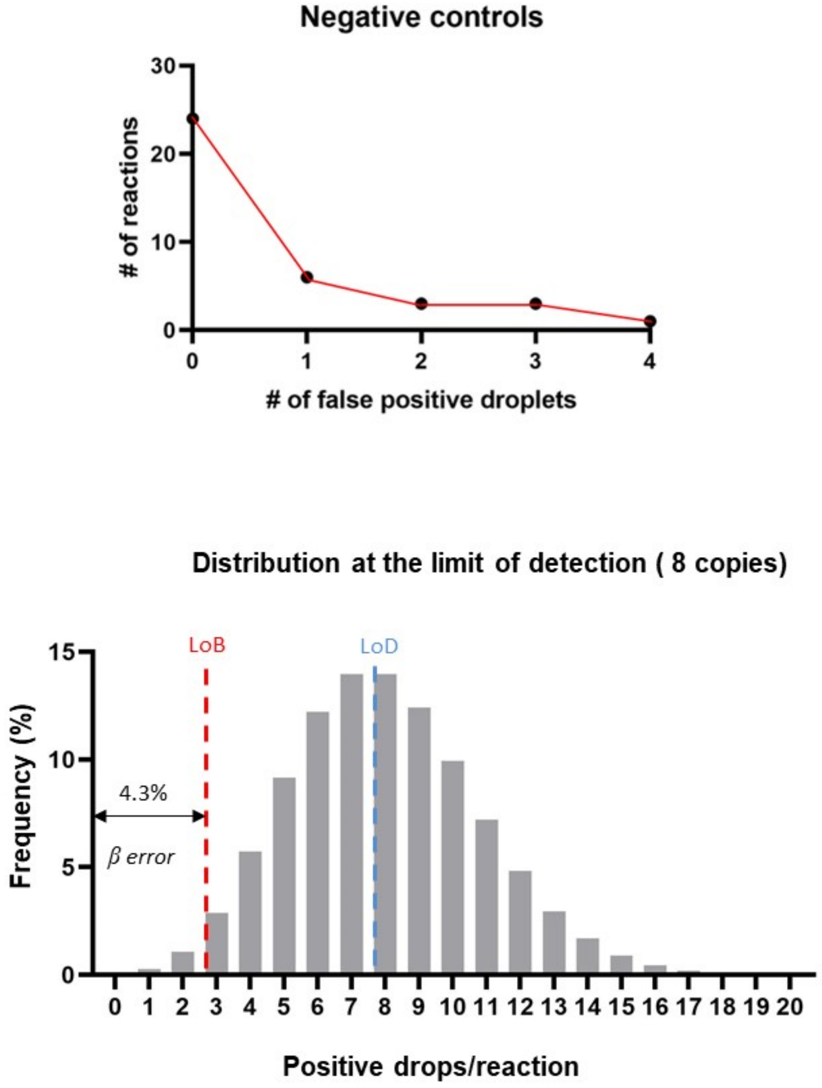

Fig S4. Our ddPCR conditions can detect 8 copies of the mutant alleles in heterogenous MEFs. The limits and confidence intervals of our assays were established by determining a limit of blank (LoB) and a lower limit of detection (LoD) using our serial dilution samples. There were 24 positive drops in a total of 38 reactions giving an average false positive rate (Afp) of 0.71, LoB of 3 drops/reaction with α error<5%. The LoD was determined by a minimum droplet number with β error<5%, which was achieved at 8 drops/reaction. These numbers were not affected by total genomic DNA copy numbers in the reaction. The bottom graph represents the theoretical Poisson distribution when a sample is prepared at the LoD (8 copies/reaction). The arrow to the left of the LoB is β error.

Fig S5

	A	B	C	D	E	F	G	H
Sample	mPer1 W448E Homo #2	mPer1 W448E Homo #2	mPer1 W448E Homo #12	mPer1 W448E Homo #12	mPer2 W419E Het #95	mPer2 W419E Het #95	mPer2 W419E Het #107	mPer2 W419E Het #107
Primer	in-out	in-in	in-out	in-in	in-out	in-in	in-out	in-in

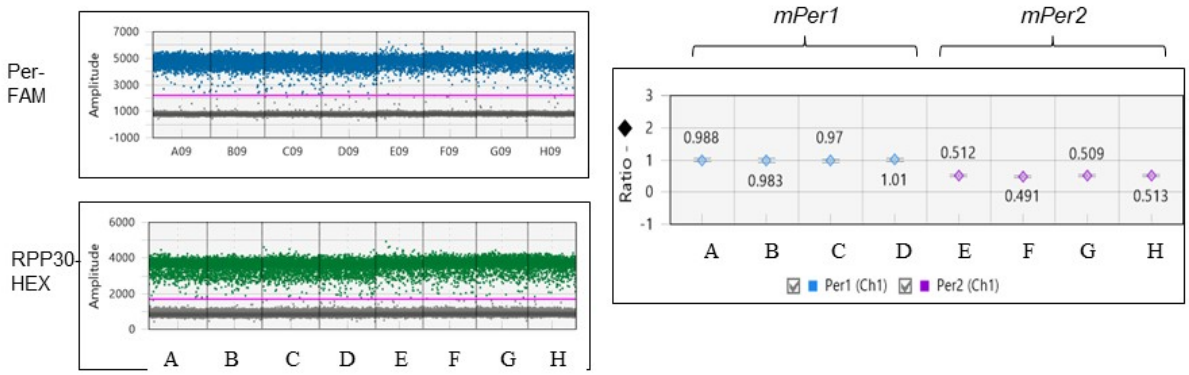

	A	B	C	D	E	F	G	H
Sample	mPer1 W448E Homo #6	mPer1 W448E Homo #6	mPer1 W448E Homo #7	mPer1 W448E Homo #7	mPer2 W419E Het #103	mPer2 W419E Het #103	mPer2 W419E Het #106	mPer2 W419E Het #106
Primer	in-out	in-in	in-out	in-in	in-out	in-in	in-out	in-in

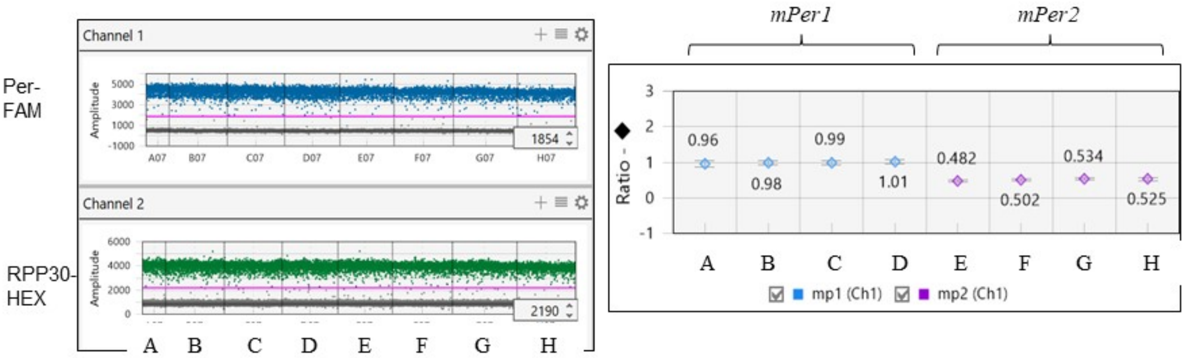

Fig S5. There are no off-target KIs in our mutant mice. To detect off-target KI mutations in our mice, ddPCR assays were performed and compared between two sets of primers, one binding inside and outside (in-out) and the other binding inside and inside (in-in) of the repair templates. Note that *mPer1/RPP30* is equal to ~1 for *mPer1* homozygous mutants, but *mPer2/RPP30* is close to ~0.5 for *mPer2* heterozygotes. Representative results are shown.

Fig S6

PCR for *mPer2*^{W419E} genotyping

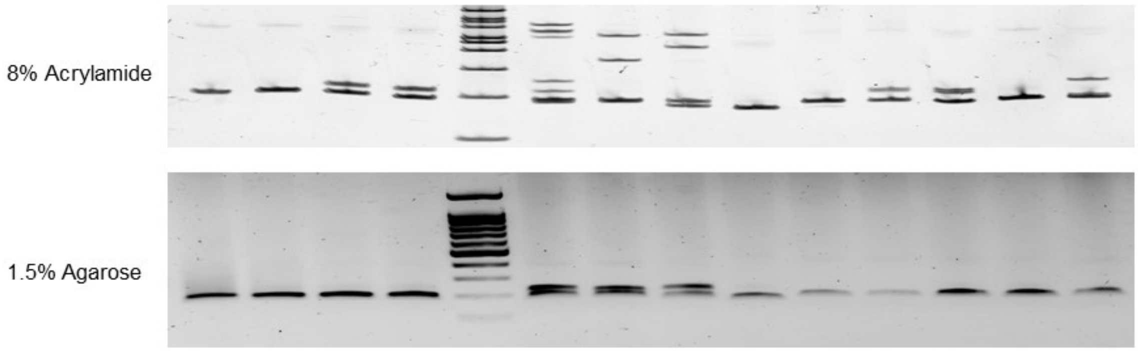

	Control				100 bp ladder	F0								
Mouse ID	F1 (#96x#97)			mixed DNA		90	92	93	94	95	96	97	98	99
mPer2 genotype	WT	P2 W419E Homo	P2 W419E / WT Het	P2 W419E Homo + WT mix		Wt / 7del	W419E / 4del	W419E / 14del	18del / Large del	W419E / large del	W419E / wt	W419E / wt	W419E / large del	W419E / 1del

Fig S6. Heteroduplex DNA mobility assay in PAGE can be used for genotyping. When genotyping amplicon samples were run on both polyacrylamide gels (PAGE) and agarose gels, multiple larger than expected bands are visible only on PAGE. We used this property for initial screening but final genotype was confirmed by enzyme digestion and Sanger sequencing. Note that the large bands are not detected on the agarose gel. Control samples were prepared from wild-type (wt) mice, *mPer2* homozygous (Homo) F1 mice, *mPer2* heterozygous (Het) F1 mice, and mixed DNA between wt and *mPer2* homo genomic samples (lane 4).

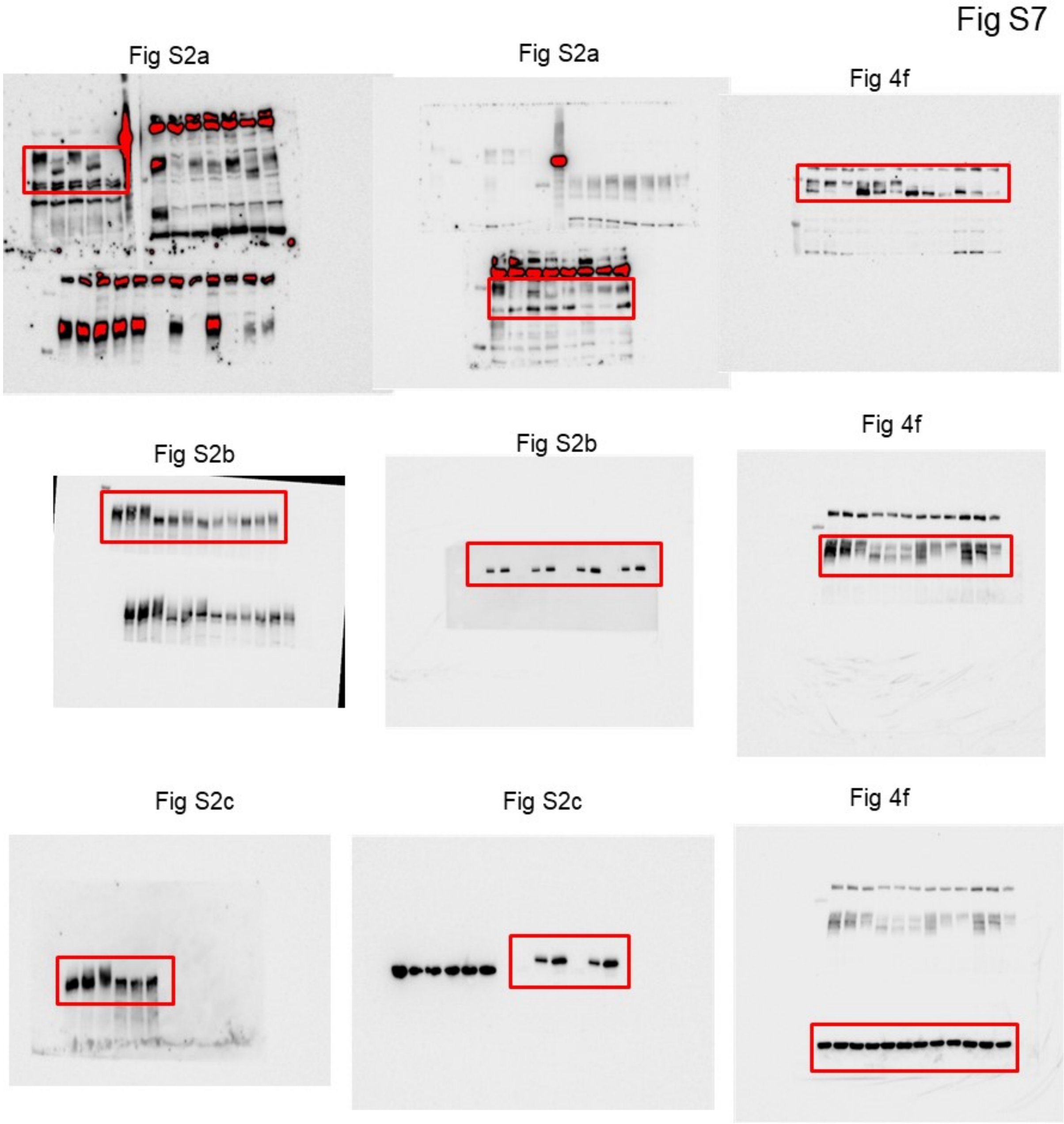

Fig S7. Uncropped images for immunoblots.

Fig S8

Fig 1a

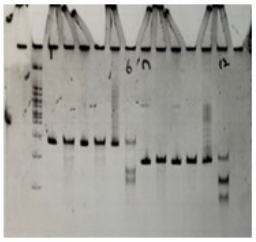

Fig 2b

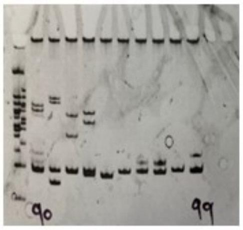

Fig 4c

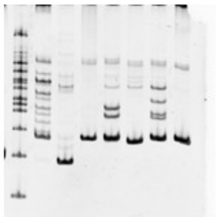

Fig 4c

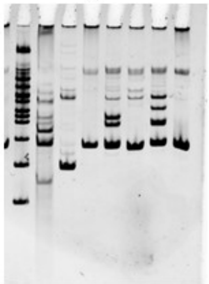

Fig 2a

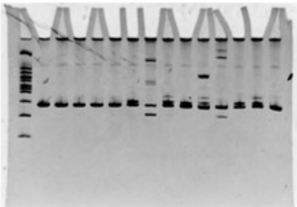

Fig 2b

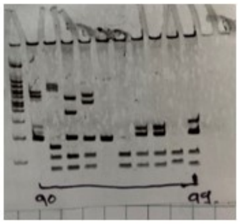

Fig 4d

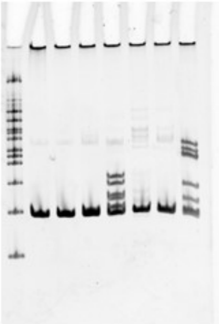

Fig 4d

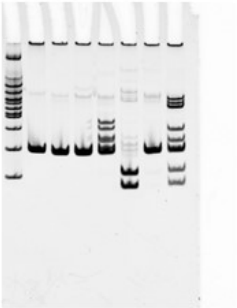

Fig 2a

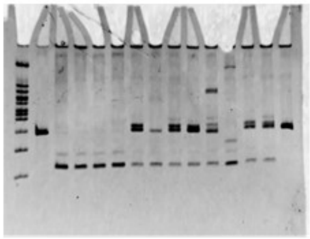

Fig S3a

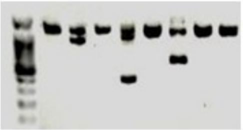

Fig S8. Uncropped images for PAGE and agarose gels.
